# Precision Medicine in Breast Cancer: Do MRI Biomarkers Identify Patients Who Truly Benefit from the Oncotype DX Recurrence Score^®^ Test?

**DOI:** 10.3390/diagnostics12112730

**Published:** 2022-11-08

**Authors:** Francesca Galati, Valentina Magri, Giuliana Moffa, Veronica Rizzo, Andrea Botticelli, Enrico Cortesi, Federica Pediconi

**Affiliations:** Department of Radiological, Oncological and Pathological Sciences, Sapienza—University of Rome, 00161 Rome, Italy

**Keywords:** early breast cancer, multiparametric MRI, MRI biomarkers, oncotype DX recurrence score, genomic assays, adjuvant therapy

## Abstract

The aim of this study was to combine breast MRI-derived biomarkers with clinical-pathological parameters to identify patients who truly need an Oncotype DX Breast Recurrence Score^®^ (ODXRS) genomic assay, currently used to predict the benefit of adjuvant chemotherapy in ER-positive/HER2-negative early breast cancer, with the ultimate goal of customizing therapeutic decisions while reducing healthcare costs. Patients who underwent a preoperative multiparametric MRI of the breast and ODXRS tumor profiling were retrospectively included in this study. Imaging sets were evaluated independently by two breast radiologists and classified according to the 2013 American College of Radiology Breast Imaging Reporting and Data System (ACR BI-RADS) lexicon. In a second step of the study, a combined oncologic and radiologic assessment based on clinical-pathological and radiological data was performed, in order to identify patients who may need adjuvant chemotherapy. Results were correlated with risk levels expressed by ODXRS, using the decision made on the basis of the ODXRS test as a gold standard. The χ^2^ test was used to evaluate associations between categorical variables, and significant ones were further investigated using logistic regression analyses. A total of 58 luminal-like, early-stage breast cancers were included. A positive correlation was found between ODXRS and tumor size (*p* = 0.003), staging (*p* = 0.001) and grading (*p* = 0.005), and between BI-RADS categories and ODXRS (*p* < 0.05 for both readers), the latter being confirmed at multivariate regression analysis. Moreover, BI-RADS categories proved to be positive predictors of the therapeutic decision taken after performing an ODXRS assay. A statistically significant association was also found between the therapeutic decision based on the ODXRS and the results of combined onco-radiologic assessment (*p* < 0.001). Our study suggests that there is a correlation between BI-RADS categories at MRI and ODXRS and that a combined onco-radiological assessment may predict the decision made on the basis of the results of ODXRS genomic test.

## 1. Introduction

Breast cancer (BC) is the most commonly diagnosed cancer in women (24.5% of malignant tumors) and the first cause of cancer-related death in the female population [1].

The evaluation of clinical, biological, and pathological factors, such as tumor burden, grading, proliferation index, receptor status, metabolic activity and nodal status, is currently required to personalize therapy in terms of surgery, endocrine treatment and/or chemotherapy (CHT).

Nowadays, routine definition of molecular subtypes of BC is based on immunohistochemical surrogates, derived from the assessment of the expression of estrogen receptor (ER), progesterone receptor (PR), human epidermal growth factor receptor 2 (HER2), and on the quantification of the proliferation index. According to commonly used immunohistochemistry, BCs can be divided into luminal-like, HER2 positive, and triple-negative subtypes [2], with ER/PR-positive tumors (luminal-like) representing about 70% of BCs [3]. In spite of the high incidence, BC mortality rate has been declining over the last few years [4] due to early cancer detection and improved treatment planning, owing to the progressive spread of techniques of molecular biology and modern breast imaging. Breast magnetic resonance imaging (MRI) is an established, non-invasive modality for the detection and characterization of BC with several well-known indications including screening of women at high-risk, preoperative staging of newly diagnosed BC and monitoring the effects of neoadjuvant therapy [5,6,7].

Biomarkers derived from multiparametric MRI (MP-MRI) examinations, including traditional T2-weighted and contrast-enhanced sequences, diffusion-weighted imaging (DWI) and MR spectroscopy, have shown to be highly correlated with histology, molecular subtypes of BC and other prognostic and predictive factors as response to treatment, risk of recurrence and overall survival [5,8,9,10,11,12,13,14,15].

Over the last few decades, the proportion of BC of early stage (including stage I to IIIa) at diagnosis has increased, reaching about 80% [16].

Women with early-stage BC are expected to have excellent survival rates; as a consequence, in these cases, the treatment is mainly based on surgical resection with or without radiotherapy. However, it is well-known that recurrences continue to occur steadily throughout 5 and 10 years follow up, especially in case of luminal cancers [17,18].

In this scenario, clinical oncologists have the hard duty to decide whether adjuvant CHT is necessary for all early-stage luminal BC or if a subgroup of these patients can omit CHT without damage.

In 2012, the Early Breast Cancer Trialists’ Collaborative Group (EBCTCG) demonstrated that adjuvant CHT can be safely omitted in about 90% of early-stage BC [19], sparing patients from burdensome side effects. Unfortunately, based on current knowledge, none of the classical clinical-pathological variables allows the identification of patients with less aggressive disease with enough confidence.

The effects of both cancer disease and CHT deeply affect patients’ lives. Indeed, they can lead to new morbidities and disabilities, causing indirect but important economic impact on the healthcare system.

The Oncotype DX Breast Recurrence Score^®^ (Genomic Health; Redwood City, CA, USA) test is a gene expression profile panel test that adds prognostic and predictive information to complementary clinical-pathological evaluation. The Oncotype DX Breast Recurrence Score^®^ (hereinafter referred to as ODXRS) test is used in clinical practice to predict the benefit of adjuvant CHT in ER positive/HER2 negative, lymph node (LN)-negative or up to three LN-positive early-stage BC disease. The international prospective and randomized trials TAILORx, RxPONDER (SWOG’s S1007) and PlanB [20,21,22,23], have demonstrated that the ODXRS test has a prognostic value and clinical utility, achieving a level of evidence and grade of recommendation of I A in the major international guidelines [24,25,26,27].

However, this genomic analysis has the main disadvantage of having a relevant cost (amounting to around EUR 3300, in Italy), limiting the use of this assay in daily practice in many countries.

Thus, the aim of this study was to combine imaging biomarkers derived from breast MP-MRI and clinical-pathological parameters to identify and correctly stratify patients who truly need the ODXRS test, in order to customize therapeutic decisions and optimize healthcare cost management in the current precision medicine era.

## 2. Materials and Methods

### 2.1. Study Population

This study was conducted according to Good Clinical Practice guidelines and obtained the approval of our institutional review board. The requirement for informed consent was waived because of the retrospective nature of the study.

A query from our institutional medical records database was performed to identify patients affected by histologically confirmed invasive BC who underwent a preoperative MP-MRI of the breast in our Institution from April 2017 to January 2018, and the ODXRS test.

Eligibility criteria for the ODXRS test were: stage I, II, or IIIa; ER-positivity, PR-positivity or both; HER2-negativity; LN-negativity or up to three LN-positivity. Patients with a history of BC or recurrent disease were excluded.

Patient data were collected using Excel 2011 (Microsoft Corporation, Redmond, WA, USA).

### 2.2. MRI Protocol

Breast MRI examinations were performed on a 3 T scanner (Discovery MR 750; GE Healthcare, Chicago, IL, USA) using an 8-channel dedicated coil, with patients in a prone position. The protocol included the following sequences (total acquisition time of about 13 min):

- Axial pre-contrast 2D FSE T2-weighted fat-suppressed sequence based on a three-point Dixon technique (IDEAL): repetition time (RT) = 11,000 ms, echo time (ET) 119 ms, echo train length (ETL) = 19, bandwidth = 62.5 kHz, matrix = 512 × 224, thickness = 3–5 mm, interval = 0.1, field of view (FOV) = 350 × 350 mm, number of excitation (NEX) = 1, scan time = 130 s.
- Axial pre-contrast diffusion-weighted echo-planar imaging (DWI-EPI) sequence: RT = 4983 ms, ET = 58 ms, bandwidth = 250 kHz, matrix = 150 × 150, slice thickness = 3–5 mm, FOV = 350 × 350 mm, NEX = 2-2-4, scan time = 230 s; DWI-EPI sequences comprised b-values of 0, 500 and 1000 s/mm^2^ and the corresponding apparent diffusion coefficient (ADC) maps were calculated automatically.
- Axial dynamic 3D spoiled GE T1-weighted fat-suppressed sequences (DISCO), based on a two-point Dixon fat-water reconstruction algorithm: flip angle = 15°, RT = 4 ms, ET = 2 ms, bandwidth = 166.7 kHz, matrix = 320 × 320, slice thickness = 1.40 mm, FOV = 340 × 340 mm, NEX = 1, performed before and 9 times after contrast agent administration (total scan time = 363 s).
- Sagittal 3D spoiled GE post-contrast T1-weighted sequence.

Post-contrast T1-weighted images were acquired after the administration of 0.1 mmol/kg (0.2 mL/kg) Gadoteridol (Prohance 279.3 mg/mL; Bracco Imaging Italia S.r.l., Milano, Italy) at a rate of 3 mL/sec. Contrast medium was power-injected through 22-gauge antecubital venous access and was followed by a 20 mL saline flush. 

Subtraction images were obtained in post-processing. 

The examination was scheduled between the 7th and the 14th day of the menstrual cycle for premenopausal women, according to current guidelines [6].

### 2.3. MR Imaging Evaluation

MR imaging sets were evaluated retrospectively by two breast radiologists (Reader 1, F.G. and Reader 2, G.M., with 10 with 5 years of experience, respectively) on a dedicated workstation, independently. The readers were aware of the aim of the study but were blinded to clinical and pathological information (including lesions’ number, their benign or malignant nature, their position and size) and to the results of the ODXRS test.

The evaluation was performed using all images available. Suspicious findings were classified according to the 2013 American College of Radiology Breast Imaging Reporting and Data System (ACR BI-RADS) lexicon [28]. All suspicious lesions (BI-RADS 4 and 5) detected were measured on early post-contrast T1-weighted sequences, and the maximum size in mm was reported. In case of multifocal/multicentric disease, the biggest lesion was considered as the index lesion and included in the statistical analysis.

T2 signal intensity was evaluated visually, and the lesions were subsequently classified as hypointense, isointense and hyperintense on the basis of the predominant signal intensity of the lesion compared with the signal intensity of the surrounding glandular tissue.

Intralesional necrosis and perilesional edema were identified as areas of high signal intensity (as high as that of water) on pre-contrast fat-suppressed T2-weighted images, localized within the lesion and around or posteriorly to the lesion, respectively.

DWI hyperintensity was evaluated qualitatively on high b-value images (b = 1000 s/mm^2^). ADC values were obtained by drawing manually a 2D region of interest of proper dimension (at least 5 mm^2^) in the center of the area of restricted diffusion on ADC maps.

Axillary LN were considered pathologic if characterized by: short axis > 1 cm, round shape, loss of the fatty hilum, and cortical thickening.

**Figure 1:**
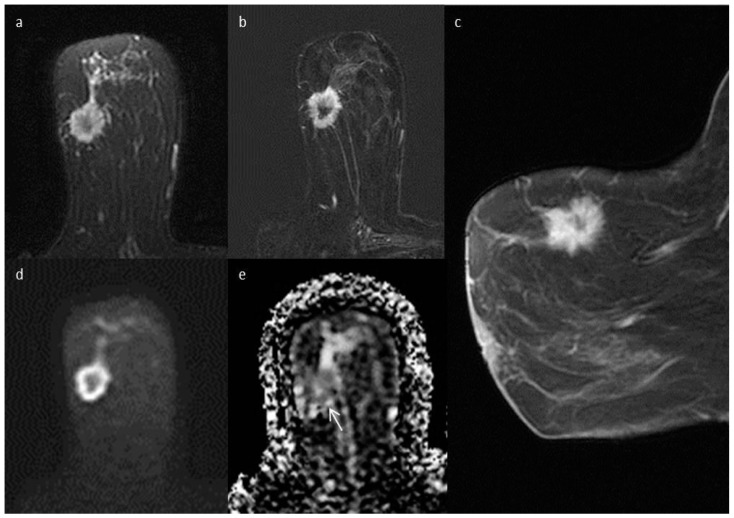
Standard breast MRI protocol. (**a**) axial fat-suppressed T2-weighted sequence shows a dishomogeneous, hyperintense mass with spiculated margins and a thin layer of perilesional edema, in the upper-outer quadrant of the right breast; (**b**) axial subtracted image and (**c**) sagittal post-Gadoteridol T1-weighted sequence show a corresponding irregular-shaped mass with spiculated margins of about 30 mm in size, characterized by heterogeneous enhancement; (**d**) axial DWI (b value = 1000 s/mm^2^) and corresponding (**e**) ADC map show the area of restricted diffusion (arrow). The finding was classified as BI-RADS 5 by both readers. Histology confirmed that the mass was an invasive carcinoma of no special type, G3, pT2, Luminal B at immunohistochemistry.

A standard MRI examination evaluated is shown in Figure 1.

### 2.4. Histopathological Analysis

Surgical specimens were analyzed by an experienced pathologist according to standardized protocols. The specimens were fixed in 10% formalin for 6–8 h, processed to obtain paraffin blocks and then cut in 5-μm-thick slices and hematoxylin-eosin stained. Tumors were classified according to the World Health Organization Classification and graded following the Nottingham Histologic Score. ER and PR status were evaluated using mouse monoclonal antibodies anti-ER alpha (6F11; Novocastra Laboratories Ltd., Newcastle upon Tyne, UK) and anti-PR (PgR-312; Novocastra Laboratories Ltd., Newcastle upon Tyne, UK). The assessment of HER2 status was carried out using a semiquantitative immunohistochemical assay (HercepTest; Dako Agilent, Santa Clara, CA, USA) and the intensity of HER2 membrane staining was scored as 0, 1+, 2+ or 3+. Equivocal results (2+) were further evaluated using fluorescence in situ hybridization for HER2 gene amplification, according to the 2013 American Society of Clinical Oncology/College of American Pathologists guidelines [29]. Ki-67 proliferation index was determined using anti-human Ki-67 monoclonal antibody MM1 (Novocastra Laboratories Ltd., Newcastle upon Tyne, UK). 

Considering immunohistochemistry, BCs were classified as luminal A-like, luminal B-like, HER2-positive and triple-negative, in accordance with the 2013 St. Gallen Consensus Conference [2].

### 2.5. Oncotype DX Breast Recurrence Score^®^ Test

All women enrolled in this study underwent ODXRS (Genomic Health Inc., Redwood City, CA, USA) tumor profiling. ODXRS is a gene-expression profiling assay based on reverse transcriptase polymerase chain reaction to evaluate mRNA expression of 16 cancer-related and 5 reference genes. The level of expression of cancer related mRNA allows for assigning a score (the so-called recurrence score) to each patient. As a result, tumors with a higher expression of proliferation genes will have a higher recurrence score and will probably show a more aggressive behavior than tumors with a low recurrence score.

Formalin-fixed, paraffin-embedded definitive surgical specimens including tumor tissue were collected for each patient and sent to Genomic Health Inc., in order to perform ODXRS test in a central laboratory. The ODXRS assay assigns a recurrence score that ranges from 0 to 100, and categorizes each patient into the following risk levels: low (recurrence score up to 15), intermediate (recurrence score from 16 to 25), and high (recurrence score of 26 or more).

In our Institution, from 2017 to 2018, the ODXRS test was conducted in all ER-positive cases, including ones with very low ER-positivity (1–10% of cells) or, on the contrary, with high ER-positivity.

### 2.6. Combined Onco-Radiologic Assessment

In a second step of the study, a combined onco-radiologic assessment was performed by a radiologist (Reader 1, F.G.) and an oncologist (V.M.) in consensus, to judge whether adjuvant CHT was necessary or not, based on clinical (including age, genetic predisposition or family history of BC, and comorbidities), pathological (including pT, according to TNM staging system [30], grading, and immunohistochemistry), and radiological (ACR BI-RADS characteristics and assessment categories) data.

A positive (need of adjuvant CHT) or negative (endocrine therapy alone) opinion was expressed and reported for each patient. The decision made by the oncologist alone on the basis of the risk level expressed by the ODXRS assay was considered as the gold standard.

### 2.7. Statistical Analysis

The Statistical Package for the Social Sciences (SPSS) 25.0 (IBM Corp., Armonk, NY, USA) was used to perform the statistical analysis. The Kolmogorov–Smirnov Z test was used to establish the normal distribution of continuous variables. Spearman’s correlation coefficient and linear regression analyses were fitted on continuous and ordinal variables to evaluate their correlation with ODXRS.

The χ^2^ test was used to investigate the association between categorical variables, including combined onco-radiologic assessment, and the decision made by the oncologist alone on the basis of the ODXRS test. Significant associations were further investigated by logistic regression.

Each analysis was performed separately for both readers (Reader 1 and Reader 2).

Inter-reader agreement between the two readers was calculated using Cohen’s κ coefficient for variables that resulted in being significantly correlated with ODXRS after regression analysis.

## 3. Results

### 3.1. Patient and Tumor Characteristics

Of the 139 patients who met the inclusion criteria, 81 were excluded for the following reasons: incomplete MRI examination (*n* = 7); ongoing neoadjuvant chemotherapy or other cancer treatments (*n* = 21); presence of BRCA mutations (*n* = 13), since differences in the distribution of ODXRS between BRCA carriers and the general population affected by BC were reported [31]; presence of breast implants (*n* = 16); core needle biopsy performed less than 14 days before MRI, in order to eliminate possible bias due to the diagnostic procedure such as the presence of a voluminous post-biopsy hematoma (*n* = 15); incomplete clinical/histological data (*n* = 9).

Therefore, a total of 58 patients were included in the study.

The Kolmogorov–Smirnov Z test demonstrated that age, Ki-67, and ADC values were normally distributed in the final population, for both readers, while the other continuous variables showed non-normal distribution. 

Mean age at diagnosis was 53.78 years (SD = 11.51, range 36–79).

Out of the 58 histologically confirmed invasive BCs, 52 lesions were classified as invasive carcinomas of no special type and 6 as invasive lobular carcinomas, according to the World Health Organization classification. Tumor grading according to Nottingham Histologic score was 1 in 7 patients (12.1%), 2 in 36 patients (62.1%), and 3 in 15 patients (25.8%).

Cancer staging at diagnosis assessed on the surgical specimens (pT) was I in 35 patients (60.3%), II in 22 (37.9%), and IIIa in 1 patient (1.7%). Thirteen patients (22.4%) had regional LN metastases at diagnosis. One patient (1.7%) had distant metastases at diagnosis (bone).

All the BCs included in the study were luminal-like according to the 2013 St. Gallen International Breast Cancer Conference classification [2].

ER expression was high (≥80%) in 55 patients (94.3%), while PR only in 4 patients (6.9%). Both ER and PR expression was ≥50% in 47 patients (81%). Ki-67 proliferation index was ≥20% in 39 patients (67.2%).

A statistically significant association was found between age and ER expression values (ρ_S_ = 0.40, *p* = 0.002).

ODXRS was low in 35 patients (60.3%), intermediate in 16 patients (27.5%), and high in 7 patients (12.1%). ODXRS had a mean value of 15.59 (SD = 8.49) and a median value of 14.0 (range 5–38).

A positive correlation was found between ODXRS and cancer staging (ρ_S_ = 0.44, *p* = 0.001) and grading (ρ_S_ = 0.36, *p* = 0.005) at diagnosis.

### 3.2. MRI

Descriptive data of MRI features are reported in Table 1.

Readers agreed that the vast majority of lesions (81%) were masses, with irregular shape and margins, while non-mass enhancements were detected in only 11 patients (19%). Reader 1 and Reader 2 found T2-weighted imaging-detectable features, such as intratumoral necrosis and peritumoral edema, in a small percentage of cases (3.4% and 27.6% for both, respectively).

A positive correlation was found between lesion size and pT (Reader 1: ρ_S_ = 0.56, *p* < 0.001; Reader 2: ρ_S_ = 0.56, *p* < 0.001). Moreover, a statistically significant association was found between lesion size and BI-RADS categories (Reader 1: ρ_S_ = 0.36, *p* = 0.005; Reader 2: ρ_S_ = 0.44, *p* < 0.001). Reader 1 assigned a BI-RADS category 4 to 41.4% of lesions and a BI-RADS category 5 to the remaining 58.6%; Reader 2 recognized 48.3% of lesions as BI-RADS 4 and 51.7% as BI-RADS 5. Inter-reader agreement was substantial concerning BI-RADS assignment (0.72, *p* < 0.001), while moderate concerning lesion size (0.53, *p* < 0.001). 

#### 3.2.1. MRI-Derived and Pathological Features vs. ODXRS

A positive correlation was found between lesion size and ODXRS (Reader 1: ρ_S_ = 0.39, *p* = 0.003; Reader 2: ρ_S_ = 0.39, *p* = 0.003) and between BI-RADS categories and ODXRS (Reader 1: ρ_S_ = 0.31, *p* = 0.016; Reader 2: ρ_S_ = 0.44, *p* = 0.01).

The association between MRI-derived and pathological features and ODXRS was assessed using the univariate and multivariate linear regression analyses. The univariate analysis proved that grading, pT, size and BI-RADS categories were significantly associated with ODXRS. The multivariate analysis confirmed that grading, pT and BI-RADS categories were significantly associated with ODXRS, while size was not (see Table 2).

#### 3.2.2. MRI-Derived and Pathological Features vs. Stratified ODXRS

Possible associations between MRI features and ODXRS stratified for level of risk of recurrence (low, intermediate and high) were evaluated using the χ^2^ test. The results are synthesized in Table 3.

#### 3.2.3. MRI vs. Post-ODXRS Assay Therapeutic Decision

After performing the ODXRS test, 43 patients (74.1%) were referred to endocrine therapy alone and 15 (25.9%) to endocrine therapy plus CHT.

In 20 patients (34.5%), the therapeutic decision was changed after performing the test.

The logistic regression analysis, performed to identify the predicting value of MRI-derived features, proved that BI-RADS categories are positive predictors of the therapeutic decision taken after performing the ODXRS genomic assay (OR = 16.10, CI 95% = 1.94–133.52, *p* = 0.01). In particular, the BI-RADS 5 category is a predictor of the post-ODXRS test decision to perform CHT in addition to endocrine therapy, in patients with early-stage BC eligible for such test. The most relevant results of χ^2^ test and logistic regression analyses are reported in Table 4.

### 3.3. Combined Onco-Radiologic Assessment

The χ^2^ test revealed the existence of a statistically significant association between the therapeutic decision taken after ODXRS genomic assay and the combined onco-radiologic assessment (*p* < 0.001), while no significant association was found between the decision based on ODXRS and the evaluation made by the oncologist alone before performing such test (*p* = 0.071).

## 4. Discussion

For decades, the important decision to add CHT to endocrine adjuvant therapy in the treatment of BC patients has been based exclusively on tumor-related, patient-related prognostic factors and on the clinical expertise and experience of breast oncologists [32].

It is now established that hormone receptor-positive early BC has commonly favorable outcomes; however, a limited group of these patients will still develop recurrent disease [17,18]. Prognostic and predictive genomic assays, such as ODXRS test, were developed to predict the benefits of adjuvant CHT in hormone receptor-positive early BC, selecting patients who would really take advantage of the treatment. In recent years, ODXRS assay has become widely adopted in clinical practice and has been included in treatment guidelines for early BC [24,25,26,27], changing de facto the management of patients affected by hormone receptor-positive early BC.

In our population, the results of the ODXRS test led to changing the decision about the use of adjuvant CHT in 20 patients (34.5%), compared with the evaluation made before performing the test. Our results are in accordance with data reported by Dieci et al. in a recent publication [33], where the use of the ODXRS test has led to a 30% rate of change in treatment decision. Furthermore, we found a positive correlation between ODXRS and cancer size, staging, and grading at diagnosis. These findings confirm existing literature too [34].

Despite the unquestionable advantages, the ODXRS test is still rather expensive. In Italy, the assay is purchased by the Public Health System or by patients for around EUR 3300, which we consider a not affordable price for general population, in spite of the demonstrated cost-effectiveness.

In our opinion, the possibility to optimize the selection of patients who will undergo the test in a clinical routine could reduce unnecessary healthcare expenses, decreasing the need for such an expensive assay. For this reason, the aim of this study was to combine imaging biomarkers derived from breast MP-MRI and clinical-pathological parameters, to identify patients who truly need an ODXRS test.

MRI has the highest sensitivity for BC detection among current breast imaging modalities [6,7] and plays an essential role in daily breast imaging practice. We are aware that breast MRI is still burdened by some limitations, including suboptimal specificity, patchy availability, and relatively high costs. Nevertheless, if our conclusions will be validated in the future, expensive genomic tests as ODXRS could be replaced by strict combined evaluations based on clinical-pathological and MRI parameters, with considerable money saving.

Breast MRI-derived biomarkers, based on imaging descriptors representative of specific underlying pathologic conditions, are extensively used in clinical practice nowadays. ACR BI-RADS is the first and most common classification system adapted to breast imaging, including MRI. The goal of ACR BI-RADS is the standardization of breast imaging terminology, providing uniformity in radiological reports and simplifying the communication between radiologists and referring physicians. Moreover, the BI-RADS system provides a final assessment category (with a corresponding approximate risk of malignancy) and recommendation for management, reducing ambiguity and differences in terms of treatment.

As far as we know, a very limited number of prior studies have evaluated the association between qualitative BI-RADS MRI descriptors and ODXRS in patients with hormone-positive early-stage BC [35,36,37,38]. In our study, a positive correlation was found between homogeneous internal enhancement of masses and low (0–15) ODXRS for both readers. This association is consistent with what has been previously reported by Grimm et al. [39], who have demonstrated that there is an inverse relationship between homogeneous enhancement and luminal B subtype (that is more aggressive and has worse relapse-free survival rates compared to luminal A subtype, as known [2]). No other BI-RADS lexicon descriptors were individually associated with ODXRS, in contrast to what has been previously demonstrated by the few similar studies available [35,36,37,38]. This unexpected result can be explained by the relatively small number of patients enrolled in this study, which was probably insufficient to reach a statistically significant correlation. Actually, the regular (round and oval) shape of masses was found exclusively in patients with low or intermediate ODXRS, as well as circumscribed margins, that were assessed more frequently in patients with low ODXRS compared with non-low ODXRS, by both readers. Similarly, the absence of perilesional edema and pathologic LN were much more frequent in patients with low ODXRS. Our opinion is that these results, that partially confirm existing literature [36,37], could reach statistical significance increasing the sample size.

A positive correlation between BI-RADS categories and ODXRS was observed, and this finding was confirmed by multivariate analysis. To our knowledge, no previous studies have had such a result. Moreover, the logistic regression analysis, performed to identify the predicting value of MRI-derived features, has proved that BI-RADS categories are positive predictors of the therapeutic decision based on the ODXRS. In particular, BI-RADS 5 category seems able to predict the decision to perform CHT in addition to endocrine therapy, in patients with early-stage BC eligible for the ODXRS test. As mentioned, the BI-RADS final assessment category represents the conclusive and most important act of every breast MRI evaluation, summarizing the main findings and providing recommendations for subsequent actions. Our results support the role of MRI-derived features as biomarkers of disease in breast cancer.

For what concerns DWI, unlike previous studies [40,41,42,43], no significant correlation was found between ADC values and ODXRS. In addition, this result could be explained by the size of the sample that was even smaller, considering that DWI sequences were absent for technical problems in part of the examinations.

The second part of our study aimed to evaluate if a combined onco-radiological assessment could aid in the selection of patients who would benefit from the execution of genomic assays, predicting ODXRS and ultimately reducing healthcare costs, limiting the need of more expensive examinations. As far as we know, this is the first study that proposes such a solution.

We firmly believe that a multidisciplinary approach is currently required, especially in the management of such a complex and heterogeneous disease as BC. For this reason, in our Institution, each patient is accurately studied in a multidisciplinary board including radiologists, surgeons, pathologists, oncologists and radiotherapists.

A statistically significant association was found between the results of combined onco-radiologic assessment and the decision based on the ODXRS. Even if preliminary, this result suggests that a decision made on the basis of an accurate evaluation of clinical, pathological and immunological characteristics of BC combined with MRI-derived imaging features classified according to ACR BI-RADS may predict the one based on the results of ODXRS.

We acknowledge that our study, even though preliminary, has several limitations: the number of patients enrolled was limited and the selected population included only women with diagnosed BC, the analysis was monocentric, and an external validation of our results was not included. Moreover, we did not use artificial intelligence systems, unlike previous studies on the same subject [44,45,46,47], since they were not available in our Institution during the study. Our objective for the future is to enlarge the population examined, possibly implementing techniques of radiogenomics.

## 5. Conclusions

Our study suggests that there is a correlation between BI-RADS categories at breast MRI and ODXRS. The combination of imaging-derived biomarkers and clinical-pathological parameters in an onco-radiological assessment may predict the decision made by the oncologist on the basis of the results of expensive and invasive genomic tests, as an ODXRS assay. According to our preliminary results, the onco-radiological assessment proposed has demonstrated to be able to accurately select patients who may really benefit from the execution of such tests, saving precious time and resources.

The replacement of ODXRS or similar assays, that cannot be currently substituted, could become effective when radiogenomic techniques, which by definition combine MRI-derived biomarkers with gene expression and clinical-pathological parameters, will be definitively validated by multi-center randomized controlled trials and integrated into clinical practice.

## Figures and Tables

**Table 1:** Descriptive MRI-derived features.

MRI Features	Reader 1	Reader 2
**Size (mm)**		
Mean (range)	20.38 (3–75)	20.19 (3–80)
**BI-RADS category**		
4	24/58	28/58
5	34/58	30/58
**T2 signal**		
Hypointensity	28/58	25/58
Hyperintensity	13/58	12/58
Isointensity	16/58	20/58
Not evaluable	1/58	1/58
**Intralesional necrosis**		
Absent	55/58	55/58
Present	2/58	2/58
Not evaluable	1/58	1/58
**Peritumoral edema**		
Absent	41/58	41/58
Present	16/58	16/58
Not evaluable	1/58	1/58
**Mass**	47/58	47/58
**Non-mass enhancement (NME)**	11/58	11/58
**Masses: Shape**		
Round	15/47	15/47
Oval	13/47	12/47
Irregular	19/47	20/47
**Masses: Margins**		
Circumscribed	11/47	10/47
Irregular	24/47	22/47
Spiculated	12/47	15/47
**Masses: Internal enhancement characteristics**		
Homogeneous	16/47	14/47
Non Homogeneous	31/47	33/47
**NME: Distribution**		
Focal	1/11	0/11
Linear	0/11	1/11
Segmental	3/11	3/11
Regional	3/11	3/11
Multiple regions	4/11	4/11
**NME: Internal enhancement patterns**		
Heterogeneous	4/11	3/11
Clumped	7/11	8/11
**DWI signal**		
Hypointensity	29/58	28/58
Hyperintensity	1/58	2/58
Isointensity	2/58	2/58
Not evaluable	26/58	26/58
**ADC value (10^−6^ mm^2^/s)**		
Mean (range)	990.04 (586–1450)	996.00 (586–1450)
**Pathologic LN**		
Absent	47/58	48/58
Present	11/58	10/58

**Table 2:** Association between pathological and MRI-derived features of the lesions evaluated and ODXRS. The analysis was performed using Spearman’s correlation. Statistically significant results were further evaluated using univariate analysis. Variables that were significant in univariate analysis were included in multivariate analysis.

	Spearman’s Correlation Coefficient		Univariate Analysis		Multivariate Analysis (Backward Stepwise Regression)	
	ρ		B (95% CI) *	B (95% CI) *	Reader 1	Reader 2
					B (95% CI) *	B (95% CI) *
**Grading**	0.362 *p* = 0.005		5.52 (2.07–8.97) *p* = 0.002		4.13 (0.73–7.53) *p* = 0.018	4.07 (0.78–7.36) *p* = 0.016
**pT**	0.441 *p* = 0.001		5.78 (1.79–9.78) *p* = 0.005		4.06 (0.18–7.94) *p* = 0.041	3.40 (−0.39–7.18) *p* = 0.077
**Lesion size**	**Reader 1**	**Reader 2**	**Reader 1**	**Reader 2**		
	0.388 *p* = 0.003	0.389 *p* = 0.003	0.15 (0.01–0.28) *p* = 0.035	0.14 (0.01–0.27) *p* = 0.033	Eliminated	Eliminated
**BI-RADS**	**Reader 1**	**Reader 2**	**Reader 1**	**Reader 2**		
	0.314 *p* = 0.016	0.440 *p* = 0.001	5.19 (0.83–9.55) *p* = 0.02	7.00 (2.9–11.11) *p* = 0.001	4.54 (0.59–8.49) *p* = 0.025	5.87 (2.06–9.68) *p* = 0.003

* B = unstandardized regression coefficient; CI = Confidence Interval.

**Table 3:** Association between pathological and MRI-derived features and ODXRS stratified for level of risk of recurrence. The analysis was performed using the χ^2^ test. Statistically significant results are bolded.

	Low (0–15)		Intermediate (16–25)		High (26–100)		*p*-Value	
**Number of Patients**	35		16		7			
**Grading**							0.058	
1	5		2		0			
2	24		10		2			
3	6		4		5			
**pT**							**0.045**	
1	26		7		2			
2	9		8		5			
3	0		1		0			
**MRI features**	**Reader 1**	**Reader 2**	**Reader 1**	**Reader 2**	**Reader 1**	**Reader 2**	**Reader 1**	**Reader 2**
**BI-RADS category**							**0.043**	**0.004**
4	19	23	4	4	1	1		
5	16	12	12	12	6	6		
**T2 signal**							0.591	0.058
Hypointensity	17	15	9	10	2	0		
Hyperintensity	8	8	2	2	3	2		
Isointensity	10	12	4	3	2	5		
Not evaluable	0	0	1	1	0	0		
**Enhancement**							0.118	0.118
Mass	31	31	12	12	4	4		
NME	4	4	4	4	3	3		
**Masses: Internal enhancement characteristics**							**0.002**	**0.026**
Homogeneous	16	13	0	0	0	1		
Non homogeneous	15	18	12	12	4	3		
**Pathologic LN**							0.118	0.344
Present	4	4	4	4	3	2		
Absent	31	31	12	12	4	5		

**Table 4:** Association between pathological and MRI-derived features and post-ODXRS therapeutic decision. Statistically significant results were further evaluated using univariate analysis. Variables that were significant in univariate analysis were included in multivariate analysis. Statistically significant results are bolded.

	χ^2^ Test		Univariate Logistic Regression		Multivariate Logistic Regression	
	*p*-Value		OR (95% CI) *	OR (95% CI) *	OR (95% CI) *	OR (95% CI) *
**Grading**	0.082					
**pT**	0.326					
**MRI features**	**Reader 1**	**Reader 2**	**Reader 1**	**Reader 2**	**Reader 1**	**Reader 2**
**BI-RADS**	0.002	<0.001	16.1 (1.94–133.52) *p* = **0.010**	23.63 (2.83–197.00) *p* = **0.003**		48.00 (4.90–469.60) *p* = **0.001**
**T2 signal** (Hypointensity vs. Non hypointensity)	0.154	0.03		4.40 (1.08–17.89) *p* = **0.038**		10.57 (1.95–57.19) *p* = **0.006**
**Mass enhancement** (Homogeneous vs. Non homogeneous)	**0.006**	0.086	Out of scale			
**Pathologic LN**	0.099	0.262				

* OR = Odds Ratio; CI = Confidence Interval.

## Data Availability

The data presented in this study are available upon reasonable request from the corresponding author.
